# Substance use and inhalation injury in adult burn patients: retrospective study of the impact on outcomes

**DOI:** 10.1186/s41038-019-0152-5

**Published:** 2019-06-04

**Authors:** Kevin M. Klifto, Luis Quiroga, C. Scott Hultman

**Affiliations:** Department of Plastic and Reconstructive Surgery, Johns Hopkins Burn Center, The Johns Hopkins Hospital, 4940 Eastern Avenue, Baltimore, 21224 USA

**Keywords:** Intubation, Smoking, Burn, Risk factors, Cocaine, Benzodiazepines, Morphine, Inhalation

## Abstract

**Background:**

Substance use, alcohol use, and smoking use have all been associated with burn injury. Few studies have investigated associations with substances, alcohol, smoking, inhalational only burns, and patient outcomes. The purpose of the study was to identify risk factors for pulmonary failure in patients suffering inhalation injury, focusing on the impact of substance, alcohol, and cigarette use.

**Methods:**

This is a single-center retrospective analysis of 115 patients admitted to the Johns Hopkins Bayview Burn Center with inhalational injury from January 1, 2010, through September 30, 2018. Patients were excluded if they were under the age of 18 years or had burn involvement of the skin > 5%. Primary outcome variables measured were if patients were intubated, length of total time intubated, substance use, alcohol use, and smoking use. Secondary outcome variables measured were types of substances used (amphetamines, barbiturates, benzodiazepines, cannabinoids, cocaine, methadone, codeine/morphine), total number of substances used, intensive care unit (ICU) length of stay (LOS), hospital LOS, secondary complications, and patient mortality. Analysis was performed with Fisher’s exact test and the Mann-Whitney *U* test. A sub-group analysis for each substance, alcohol, smoking, and control sub-group was compared to its respective sub-group without substance, alcohol, smoking, and control within the no intubation and intubation group. A sub-group analysis of substance use, alcohol use, smoking use, and control was further analyzed with binomial logistic regression within the intubation group.

**Results:**

Following inhalation injury, 50/115 (43%) patients required intubation. Forty-two of the 50 (84%) patients intubated had substance use (*p* < 0.001). Thirty-one of the 50 (62%) patients intubated had history of smoking (*p* = 0.038). Among the specific substances used, 26/50 (52%) patients intubated were using benzodiazepines (*p* < 0.001) and 7/50 (14%) patients were using cocaine (*p* = 0.022). The lengths of intubation, ICU LOS, and hospital LOS with no substance use were shorter than with substance use (*p* < 0.001). Following the adjusted sub-group analysis, patients with substance use (odds ratio (OR) 6.4, 95% confidence interval (CI) [2.5–16.3]; *p* < 0.001) and smoking use (OR 2.5, 95% CI [1.2–5.1]; *p* = 0.013) were more likely to be intubated on admission than those without substance or smoking use.

**Conclusions:**

In patients admitted with an inhalational injury with less than or equal to 5% external burns, the presence of a substance and smoking use on admission provides a further risk of intubation and respiratory compromise. Substance use on admission poses a greater risk of longer intubation, ICU LOS, and hospital LOS. A higher potential for substance use should be suspected in this patient population with prompt treatment.

## Background

Substance use, alcohol use, and smoking use have all been associated with burn injury [1–6]. Early excision and grafting of a deep burn is the standard of care, but the presence of inhalation injury with pulmonary failure can delay wound management and skin closure [7].

Inhalation injury is defined as the trauma to the respiratory tract following the inhalation of smoke and/or chemical products of combustion [7–9]. In isolation, it has been shown to be associated with long-term pulmonary failure [10]. These injuries are very serious and at a minimum require consultation at a regional burn center [7]. Smoking, alcohol, and other substances through intoxicating effects delay patient reactions and reduce the ability of proper assessment [1, 2, 5, 6]. Alcohol possesses further immunosuppressant effects that can predispose hospitalized patients to secondary infections and increased mortality [2, 5, 11].

Currently, it remains difficult to judge the extent of an inhalation injury on admission and predict patient outcomes [8, 9]. In the majority of cases, subjective findings and physical exam findings can be used to preliminarily diagnose inhalation injury [9, 12]. Preliminary diagnosis is confirmed with either fiberoptic bronchoscopy (FOB), chest computed tomography (CT) scans, carboxyhemoglobin measurement, radionuclide imaging, and/or pulmonary function testing; [7, 12–14] however, substance use, alcohol use, and/or substance use can complicate the clinical picture in these high-risk patients [7].

Few studies have investigated associations with substances, alcohol, smoking, inhalational only burns, and patient outcomes. Thus, this study aims to look at the data gathered at the Johns Hopkins Bayview Burn Center on the outcomes of patients diagnosed with inhalation injury to develop a better understanding of patterns seen in patients with these injuries based on this diagnosis with concurrent substance, alcohol, and/or smoking use. The purpose of the study was to determine whether substance, alcohol, and smoking use has an impact on the outcome of patients admitted with inhalation injury.

## Methods

### Study design

This study is a single-center retrospective medical record review analysis of a cohort of 115 patients admitted to the Johns Hopkins Bayview Burn Center with inhalational injury from January 1, 2010, through September 30, 2018. Patients were included if they had a hospital diagnosis of inhalation injury. Inhalation injury was defined by subjective findings (history of exposure to flames, smoke, chemicals, exposure in a closed space, loss of consciousness) and physical exam findings (facial burns, singed facial or nasal hair, soot or carbonaceous material on the face or in the sputum, and signs of airway obstruction). The preliminary diagnosis was confirmed with FOB (erythema, edema, deposits, and/or obstruction). Patients were excluded if they were under the age of 18 years or had burn involvement of the skin greater than 5% total body surface area (TBSA). Burns greater than 5% were excluded to minimize confounding variables. The study was approved by the institutional review board at The Johns Hopkins Hospital IRB#00187271.

### Outcomes analyzed

Primary outcomes measured were intubation rate defined as no intubation (observed with no signs of airway compromise or respiratory distress) or intubation (intubated prior to hospital arrival, loss of consciousness, physical exam: findings of airway compromise, edema, shortness of breath) not necessarily requiring mechanical ventilation, length of intubation measured in days, substance use, alcohol use, and smoking use. Substance use was defined as a positive urine drug screen at the time of admission. Alcohol use was defined as a detectable blood alcohol concentration (BAC) greater than 30 mg/dL on admission. Smoking use was defined as either a current smoker or quit within the last 6 months. Patients were assessed by current use on admission for substances and alcohol, not by past medical histories.

Secondary outcomes measured were types of substances used, determined by seven possible substances on urine drug screen (amphetamines, barbiturates, benzodiazepines, cannabinoids, cocaine, methadone, codeine/morphine); total number of substances used; length of total time intubated in days; intensive care unit (ICU) length of stay (LOS) in days; hospital LOS in days; secondary complications (hospital-acquired infection, ventilator-associated events, hospital-acquired pressure injury, deep vein thrombosis (DVT)/pulmonary embolism (PE)); and patient mortality.

### Data management

The patient data was entered and stored into a hospital-monitored, encrypted, password-protected virtual SAFE desktop, and accessed within the Johns Hopkins Bayview Burn Center system.

### Statistical analysis

Statistical analysis was performed to compare factors associated with patient intubation after sustaining inhalational injury. Patients were grouped by either no intubation or intubation. We used the Fisher’s exact test for categorical variables and the Mann-Whitney *U* test for continuous variables, based on the nonparametric distribution of population data. One sub-group analysis of substance use, alcohol use, smoking use, and controls was further analyzed for patients in the no intubation and intubation groups to compare outcomes. Substance use, alcohol use, smoking use, and control outcomes were then compared to each other using logistic regression for categorical variables or linear regression for continuous variables. The second sub-group analysis of substance use, alcohol use, smoking use, and controls were further analyzed with binomial logistic regression within the intubation group. All analyses were performed with IBM SPSS Version 25.0 (IBM Corporation, Redmond, Washington).

## Results

### Demographics

Table 1 contains our patient demographics stratified into no intubation and intubation groups. Overall (*n* = 115, median age 54 years, range 18–94 years), 65/115 patients were not intubated (median age 52, range 18–89), and 50/115 patients were intubated (median age 57, range 18–94) on admission to the burn center for inhalation injury (*p* = 0.229). There were 38 males and 27 females not intubated, and 28 males and 22 females intubated (*p* = 0.634). Fifteen of the 115 (13%) patients tested positive for both substance and alcohol use.

**Table 1:** Patient demographic comparisons between the no intubation and intubation groups

Variables	Total (*n* = 115)	No intubation (*n* = 65)	Intubation (*n* = 50)	*P* value
Age, median, (range)	54 (18–94)	52 (18–89)	57 (18–94)	0.229
Male, *n* (%)	66 (57)	38 (58)	28 (56)	0.634
Alcohol use, *n* (%)	23 (20)	10 (15)	13 (26)	0.153
Substance use, *n* (%)	70 (61)	28 (43)	42 (84)	< 0.001
Amphetamines	0	0	0	–
Barbiturates	0	0	0	–
Benzodiazepines	32	6	26	< 0.001
Cannabinoids	12	6	6	0.768
Cocaine	8	1	7	0.022
Methadone	8	2	6	0.138
Codeine/morphine	23	12	11	0.999
Total number of substances positive on urine toxicology screen	84	28	56	< 0.001
Smoking use, *n* (%)	65 (57)	34 (52)	31 (62)	0.038

### Substance use

Seventy patients (61%) had substance use. Of these 70/115 patients, 42/50 were intubated (*p* < 0.001). Among the specific substances used, 26/50 (52%) patients intubated were using benzodiazepines (*p* < 0.001) and 7/50 (14%) patients were using cocaine (*p* = 0.022) (Table 1). The median length of intubation with no substance use was 1 day, range 0.8–23 days, and substance use was 1 day, range 0.7–27 days (*p* < 0.001). Median ICU LOS for the intubation group with no substance use was 2.5 days, range 0–47 days, and substance use 3 days, range 0–379 days (*p* < 0.001). Median hospital LOS for the intubation group with no substance use was 3.5 days, range 0–29 days, and substance use 4 days, range 1–392 days (*p* = 0.001). The total number of substances positive on urine toxicology screen in the no intubation group was 28 and in the intubation group 56 (*p* < 0.001). Mortality was not increased in patients with a positive toxicology screen (*p* = 0.519). Following the adjusted sub-group analysis, there were no significant differences for substance use in the no intubation group and intubation group compared to no substance use (Tables 2 and 3). Patients with substance use (odds ratio (OR) 6.4, 95% confidence interval (CI) [2.5–16.3]; *p* < 0.001) were more likely to be intubated on admission than those without use suffering from inhalation injury (Table 4).

**Table 2:** Sub-group analysis of outcomes associated with substance, alcohol, smoking, and controls in the no intubation groups

Variables	Substance (*n* = 28)	Alcohol (*n* = 10)	Smoking (*n* = 34)	Controls (*n* = 20)	*P* value
Hospital-acquired infection	0	0	0	0	–
Ventilator-associated events	0	0	0	0	–
Hospital-acquired pressure injury	–	–	–	–	–
DVT/PE	0	0	0	0	
ICU LOS, median (range), days	1 (0–20)	1 (0–20)	1 (0–8)	0.5 (0–5)	NS
Hospital LOS, median (range), days	2 (0–355)	1.5 (0–8)	1 (0–30)	1 (0–29)	NS
Overall mortality, *n* (%)	0 (0)	0 (0)	0 (0)	0 (0)	–

*DVT* deep vein thrombosis, *PE* pulmonary embolism, *ICU* intensive care unit, *LOS* length of stay, *NS* not significant

**Table 3:** Sub-group analysis of outcomes associated with substance, alcohol, smoking, and controls in the intubation groups

Variables	Substance (*n* = 42)	Alcohol (*n* = 13)	Smoking (*n* = 31)	Controls (*n* = 4)	*P* value
Hospital-acquired infection	3	1	3	1	NS
Ventilator-associated events	2	1	0	1	NS
Hospital-acquired pressure injury	–	–	–	–	–
DVT/PE	0	0	1	1	NS
ICU LOS, median (range), days	3 (0–379)	3 (0–24)	3 (0–379)	1 (0–7)	NS
Hospital LOS, median (range), days	4 (1–392)	5 (1–24)	4 (0–392)	5 (0–29)	NS
Overall mortality, *n* (%)	2 (5)	1 (8)	1 (3)	0 (0)	NS

*DVT* deep vein thrombosis, *PE* pulmonary embolism, *ICU* intensive care unit, *LOS* length of stay, *NS* not significant

**Table 4:** Sub-group analysis of adjusted outcomes with alcohol, substance, smoking, and controls in intubation groups

Intubation (*n* = 50)	OR	95% CI	*P* value
Alcohol use	1.6	0.6–4.2	0.354
Substance use	6.4	2.5–16.3	< 0.001
Smoking use	2.5	1.2–5.1	0.013
Controls	0.1	0.03–0.3	< 0.001

*OR* odds ratio, *CI* confidence interval

### Alcohol use

Twenty-three (20%) patients had alcohol use. Of these 23/115 patients, 13/50 were intubated (*p* = 0.153) (Table 1). The median length of intubation with no alcohol use was 1 day, range 0.7–27 days, and alcohol use was 0.8 days, range 0.8–26 days (*p* = 0.143). Median ICU LOS for the intubation group with no alcohol use was 3 days, range 0–379 days, and alcohol use 3 days, range 0–24 days (*p* = 0.272). Median hospital LOS for the intubation group with no alcohol use was 4 days, range 0–392 days, and alcohol use 5 days, range 1–24 days (*p* = 0.339). Mortality was not increased in patients with an elevated BAC on admission (*p* = 0.385). Following the adjusted sub-group analysis, there were no significant differences for alcohol use in the no intubation group and intubation group compared to no alcohol use (Table 2 and Table 3). Patients with alcohol use (OR 1.6, 95% CI [0.6–4.2]; *p* = 0.354) were as likely to be intubated on admission as those without use suffering from inhalation injury (Table 4).

### Smoking use

Sixty-five (57%) patients had smoking use. Of these 65/115 smokers, 31/50 were intubated (*p* = 0.038) (Table 1). The median length of intubation with no smoking use was 1 day, range 0.8–27 days, and smoking use was 1 day, range 0.7–10 days (*p* = 0.052). Median ICU LOS for the intubation group with no smoking use was 2 days, range 0–27 days, and smoking use 3 days, range 0–379 days (*p* = 0.02). Median hospital LOS for the intubation group with no smoking use was 7 days, range 0–355 days, and smoking use 4 days, range 0–392 days (*p* = 0.588). Mortality was not increased in smokers (*p* = 0.999). Following the adjusted sub-group analysis, there were no significant differences for smoking use in the no intubation group and intubation group compared to no smoking use (Table 2 and Table 3). Patients with smoking use (OR 2.5, 95% CI [1.2–5.1]; *p* = 0.013) were more likely to be intubated on admission than those without use suffering from inhalation injury (Table 4).

### Complications

A total of 10/115 secondary complications were observed in the study population, all occurring in the intubation group (Table 5). Five from hospital-acquired infections, three from ventilator-associated events, zero reported hospital-acquired pressure injuries, and two DVT/PEs. Two mortalities were observed, both within the intubation group. One patient was found unconscious trapped in a porch fire, and the other suffered from a hospital-acquired infection and ventilator-associated event. Both patients had a positive toxicology screen on admission. The unconscious patient tested positive for benzodiazepines and had a BAC of 292 mg/dL, the patient suffering hospital complications tested positive for codeine/morphine. Tables 2 and 3 compare outcomes for substances, alcohol, smoking, and controls, between the no intubation group and intubation group, respectively.

**Table 5:** Associated outcomes between the no intubation and intubation groups

Variables	Total (*n* = 115)	No intubation (*n* = 65)	Intubation (*n* = 50)	*P* value
Complications, *n* (%)	10 (9)	0 (0)	10 (20)	< 0.001
Hospital-acquired infection	5	0	5	0.014
Ventilator-associated events	3	0	3	0.079
Hospital-acquired pressure injury	0	0	0	–
DVT/PE	2	0	2	0.187
ICU LOS, median (range), days	1 (0–379)	1 (0–20)	3 (0–379)	< 0.001
Hospital LOS, median (range), days	1 (0–392)	1 (0–355)	4 (0–392)	< 0.001
Overall mortality, *n* (%)	2 (2)	0 (0)	2 (4)	0.187

*DVT* deep vein thrombosis, *PE* pulmonary embolism, *ICU* intensive care unit, *LOS* length of stay

## Discussion

The results show that patients admitted to the burn center for suspected inhalation injury are at a higher risk of being intubated and mechanically ventilated if they are a current smoker or have quit smoking within the past 6 months, and/or if they have substance use. This information may provide future guidance for managing inhalation injuries. A positive toxicology screen on admission was associated with intubation. We speculate that the results of a positive urine toxicology screen on admission may predict patients at risk for further respiratory compromise. This knowledge may help to mobilize the appropriate resources. Our study revealed that benzodiazepine and cocaine use were risk factors for intubation.

Benzodiazepines are one of the most commonly prescribed substances that can decrease lung function [15, 16]. Long-acting subclasses of these medications can have half-lives that approach 250 h, extending the pharmacological effects on the lungs for weeks [15]. They relax pharyngeal skeletal muscles to narrow the airway, relax intercostal skeletal muscles to decrease lung expansion, and relax smooth muscles of the lungs to decrease minute ventilation, respiratory rate, and tidal volume [16]. The lung capillaries try to compensate by constricting and shunting blood flow to areas with higher perfusion. This process further impairs lung function by inducing hypoxic pulmonary vasoconstriction [17]. Although none of our patients tested positive for barbiturates, they have a similar mechanism of action to benzodiazepines. A toxicology screen may help by allowing healthcare professionals to monitor these patients more closely and provide earlier supportive care.

Urine tests were positive for cocaine for approximately 1 to 2 days. The relatively short half-life of 6 h allows us to assume these patients used this substance around the time of the event [18]. Pulmonary toxicity is uncommon, unless the substance has been inhaled and manifests as “crack lung” [19]. This can induce barotrauma, bronchoconstriction, noncardiogenic pulmonary edema, pneumothorax, pneumomediastinum, and worsening of preexisting lung disease [18]. If cocaine is chronically inhaled, reactive airway disease and tracheal stenosis can develop [20]. We must be aware cocaine administration can occur by many routes, and a patient with inhalation injury testing positive for cocaine metabolites may be at a higher risk for respiratory failure.

The two prospective studies by Silver et al. [5] and Davis et al. [2] both concluded an elevated blood alcohol content on admission is associated with poorer outcomes, larger burns, and more inhalational injury. Fluid requirements, duration of mechanical ventilation, ICU LOS, hospital LOS, and mean hospital charges were all increased in patients with alcohol abuse. Our study, by excluding the large burn patients, suggests that those outcomes are mainly caused by the burn and not inhalation injury.

Smoking is a well-known cause of lung injury and the leading cause of death from fire [21]. The chemicals inhaled by smoking cigarettes deposit in the lungs and induce inflammation. Electronic cigarettes are odorless methods of smoking that still induce lung injury [22]. Aerosolized nicotine has been found to cause inflammation by recruiting inflammatory markers that produce pulmonary edema [22, 23]. The absence of the scent of cigarettes should not eliminate the possibility of a smoker suffering an inhalation injury. Smoking is a risk factor that has historically predisposed patients to burn injuries and intubation [1, 8–10, 24].

Overall mortality in burn patients with inhalation injury has been reported at 41.5% compared to a value of 7.2% in patients not suffering inhalation injury. Age and TBSA of burns are predictors of increased mortality that are incorporated in this high reported mortality of 41.5% [25]. The 2% mortality rate we reported may be lower due to less than 5% TBSA and the expertise of management at a national burn center. Acute respiratory distress syndrome and rates of mechanical ventilation are higher in the literature with larger TBSA burns [26, 27].

Limitations of our study relate to the small sample size and retrospective study design. The sample size did not allow for a normal distribution; therefore, we had to use Fisher’s exact test instead of Pearson’s chi-square test for categorical data. Similarly, we had to use the Mann-Whitney *U* test instead of the two-sample independent *t* test for continuous data. The incidence of inhalation injuries has been reported as 2.2% in patients suffering less than 20% TBSA [28]. Our 115 inhalation injury patients with less than 5% TBSA burns over the time period of 8 years was a reasonable population number for our study. We analyzed data from a single-center, limiting the generalizability to the worldwide population. Our population may represent a higher level of alcohol, substance, and smoking use compared to other burn centers. Baltimore City reported 232 substance and alcohol intoxication deaths in 2009, 172 intoxication deaths in 2010, and 165 intoxication deaths in 2011 [29]. Opioid use was measured by codeine and morphine metabolites. Synthetic and semi-synthetic opioids do not routinely test positive by this screening method. This may explain why we did not see a difference in the opioid and non-opioid group of patients intubated.

This was the first study to evaluate sub-categories of substance use, alcohol use, and smoking use as risk factors for intubation in a population with less than 5% TBSA burns. By isolating a group of patients with just inhalation injury, it allowed us to determine risk factors and predictive outcomes specific to this location of burn.

## Conclusion

In patients admitted with an inhalational injury with less than or equal to 5% external burns, the presence of substance and smoking use on admission provides a further risk of intubation and respiratory compromise. Substance use on admission poses a greater risk of longer intubation, ICU LOS, and hospital LOS. A higher potential for substance use should be suspected in this patient population with prompt treatment. This important finding may delay surgical skin grafting and contribute to poor outcomes in patients with external burns.

## Abbreviations

BAC: Blood alcohol concentration
DVT: Deep vein thrombosis
FOB: Fiberoptic bronchoscopy
ICU: Intensive care unit
LOS: Length of stay
PE: Pulmonary embolism
TBSA: Total body surface area
